# Metabolomics analysis reveals a protective effect of hydroxycitric acid on calcium oxalate-induced kidney injury

**DOI:** 10.22038/ijbms.2024.75089.16343

**Published:** 2024

**Authors:** Pei Cao, Yaqian Li, Zhiqing Zhang

**Affiliations:** 1 Department of Pharmacy, The Second Hospital of Hebei Medical University, Shijiazhuang, 050051, P. R. China

**Keywords:** Calcium oxalate Hydroxycitric, acid Metabolomics, Renal injury, UHPLC-Q-TOF-MS/MS

## Abstract

**Objective(s):**

Prior research has indicated that hydroxycitric acid (HCA) can impede the formation of calcium oxalate (CaOx) crystals, yet the specific mechanisms underlying its therapeutic effects remain unclear. In this study, we delved into the protective effects of HCA against glyoxylate-induced renal stones in rats and sought to elucidate the underlying metabolic pathways.

**Materials and Methods:**

Forty rats were randomly assigned to five groups: control group, model group, L-HCA-treated group, M-HCA-treated group, and H-HCA-treated group. Von Kossa staining was conducted on renal sections, and blood urea nitrogen and serum creatinine were determined by biochemical analysis. Meanwhile, body weight and urine volume were also measured. We subjected urine samples from the rats to analysis using ultra-performance liquid chromatography-quadrupole time-of-flight mass spectrometry. Next, we employed a metabolomic approach to scrutinize the metabolic profiles of each group.

**Results:**

HCA significantly reduced blood urea nitrogen and serum creatinine, and increased body weight and urine volume. It also reduced CaOx crystal deposition. A total of 24 metabolites, exhibiting a significant reversal pattern following HCA administration, were identified as urine biomarkers indicative of HCA’s preventive effects against CaOx crystal-induced renal injury. These metabolites are primarily associated with glycine, serine, and threonine metabolism; phenylalanine metabolism; tricarboxylic acid cycle; taurine and hypotaurine metabolism; and tryptophan metabolism.

**Conclusion:**

It was demonstrated that HCA has a protective effect against CaOx crystal-induced kidney injury in rats by modulating various metabolic pathways. Additionally, results suggest that HCA holds promise as a potential clinical therapeutic drug for both the prevention and treatment of renal stones.

## Introduction

Renal stone disease is characterized by recurrent episodes of renal colic and hematuria, which not only impose a significant burden on the affected individuals’ quality of life but also pose a substantial community health threat (1, 2). Various factors, including environmental factors, ethnicity, metabolic disorders, and dietary habits, can influence the incidence of this condition, which ranges globally from 1% to 20%, with an average of 10% (3-6). Numerous epidemiological studies have indicated that the development of kidney stones increases the risk of chronic kidney disease (7, 8).

Calcium oxalate (CaOx) constitutes the primary component in 70% of all kidney crystals or stones (9, 10). Therefore, addressing CaOx crystal kidney disease is considered crucial in the prevention and treatment of renal stones. A derivative of citric acid known as hydroxycitric acid (HCA) has been studied in previous research, demonstrating its ability to enhance renal CaOx crystal deposition and reduce urinary calcium excretion in a rat model of oxalate stone formation (11-13). Despite this promising evidence, comprehensive metabolomics investigations into the potential anti-kidney stone effects of HCA and the underlying mechanisms have yet to be conducted.

Metabolomics, a rapidly evolving field within systems biology, involves an in-depth examination of endogenous metabolic processes within complex biological systems (14-16). This approach is increasingly being used in research to better understand the therapeutic mechanisms of medications (17-20). In this study, we explored changes in urine metabolomic profiles in a rat model of CaOx crystal-induced kidney damage and assessed the potential renoprotective effects of HCA using a metabolomics-based approach.

## Materials and Methods

***Chemicals and reagents***

Formic acid was obtained from Dikma (Foothill Ranch, CA, USA), while high-performance liquid chromatography grade methanol and acetonitrile were purchased from TEDIA (Fairfield, OH, USA). Deionized water was sourced from Wahaha Corporation (Hangzhou, China), and HCA was supplied by Sigma-Aldrich (St. Louis, MO, USA). All other compounds used were of analytical grade.

***Animals and treatments***

The SD rats (200-220 g) were provided by Hebei Medical University in Shijiazhuang, China, and they underwent a one-week acclimation period. During this time, the rats were housed in a carefully controlled environment with a 12-hr light/dark cycle, at 25 ^°^C, and a relative humidity of 55%. They were given unrestricted access to regular food and water. All experimental procedures were conducted in accordance with ethical standards approved by the Laboratory Animal Research Ethics Committee of Hebei Medical University.

A total of 40 rats were randomly divided into five groups, which included the following: the blank group (control group)(*n*=8), glyoxylate group (model group)(*n*=8), glyoxylate solution+low-dose HCA (L-HCA-treated) group (*n*=8), glyoxylate solution+middle-dose HCA (M-HCA-treated) group (*n*=8), glyoxylate solution + high-dose HCA (H-HCA-treated) group (*n*=8). Glyoxylate was administered intraperitoneally to all rats at a dosage of 100 mg/kg, except for the control group, and this was done once daily for a period of 7 days. All HCA-treated were administered HCA at varying dosages of 0.25, 0.5, and 1 g/kg (low, middle, and high dose) via intragastric gavage.

After the final gavages, all rats were housed in metabolic cages for a 24-hour period to collect urine samples. Blood samples were obtained through abdominal aortic collection, and kidney tissues were removed and fixed in 10% neutral-buffered formalin for subsequent analysis. Serum samples were obtained by centrifugation at 4,000 rpm and 4 ^°^C for 5 min. All urine and serum samples were promptly stored at -80 ^°^C for further analysis.

***Measurements of biochemical parameters and histopathology***

Serum creatinine and blood urea nitrogen levels were measured using assay kits obtained from Nanjing Jiancheng Bioengineering Institute (Nanjing, China). For histological analysis, organ tissues were embedded in paraffin and preserved in 10% buffered formalin. Kidney tissue samples that had been paraffin-embedded were prepared by cutting them into 3-4 µm slices and subsequently staining them with Von Kossa. Photographs of randomly selected regions from each segment were captured using a light microscope for further examination.

***Sample preparation***

Before conducting the analysis, a 100 μl aliquot of the respective sample was thawed at 4 ^°^C, subjected to centrifugation at 13,000 rpm for 15 min, and the liquid was filtered using a 0.22 μm microporous membrane. The filtered samples were subsequently diluted with distilled water at a 1:1 ratio and vigorously mixed by vortexing. Following this, the prepared samples were transferred into sampling vials for ultra-high performance liquid chromatography (UHPLC) coupled with quadrupole-time-of-flight mass spectrometry analysis. To generate quality control (QC) samples, equal portions of all urine samples were combined.

***Analysis of urine samples***

Urine metabolite profiling was conducted using a UHPLC system (Shimadzu, Kyoto, Japan) coupled with a hybrid quadrupole tandem TOF-MS (AB SCIEX, Framingham, MA, USA). Chromatographic separations were carried out at 30 ^°^C on an ACQUITY UPLC BEH C18 column (2.1 mm×150 mm, 1.7 μm; Waters, Manchester, UK). The mobile phase consisted of 0.1% aqueous formic acid (A) andacetonitrile modified with 0.1% formic acid (B). The optimized UHPLC elution conditions were as follows: 0-1 min, 5% B; 1-10 min, 5%-10% B; 10-25 min, 10%-40% B; 25-29 min, 40%-95% B; 29-30 min; 95% B. The post-time was set at 5 min for system equilibration. The flow rate was maintained at 0.3 ml/min, and the injection volume was 4 μl. The autosampler was kept at 4 ^°^C.

The electrospray ionization source was operated in both positive and negative modes. In positive ion mode, the collision energy and declustering potential were set at 30 V and 50 V, respectively, while in negative ion mode, the collision energy and declustering potential were set at -30 V and -50 V, respectively. The source temperature was adjusted to 350 ^°^C, and ion source gas 1, ion source gas 2, and curtain gas were set at 55 kPa, 55 kPa, and 35 kPa, respectively. Nitrogen was employed as the source gas. The mass spectrum was acquired in profile mode with a range of 50 to 1,100 *m*/*z* and an accumulation time of 0.2 sec. Furthermore, the MS and MS/MS were automatically calibrated using an automated calibration supply system.

***Injection sequence and method validation***

The metabolomics sequence followed the layout as outlined below: to ensure the robust stability and reproducibility of the overall chromatographic system, six injections of QC samples were made initially (21). Subsequently, injections for the different groups were randomized. After every set of five samples, a QC sample was injected to additionally evaluate retention time shifts and mass accuracy, which were maintained within a tolerance of less than 5 ppm (22).

***Data processing and multivariate statistical analysis***

Data processing was carried out using Progenesis QI v2.3 software (Umetrics, Umeaa, Sweden), which involved peak alignment, normalization, peak picking, and deconvolution. Multivariate statistical analysis of the preprocessed data was conducted using Simca-P v14.1 software (Waters). Metabolite statistical analysis was performed through analysis of variance. Our results were analyzed using both unsupervised principal component analysis (PCA) and orthogonal partial least squares discriminant analysis (OPLS-DA). PCA, a mathematical technique for dimension reduction, helped extract comprehensive variables closely representing the original ones. The collective pattern of samples served as input for PCA, indicating whether substantial differences existed between groups. OPLS-DA, on the other hand, was a supervised discriminant analysis model used to investigate variations in urine metabolites among different populations. It also generated variable importance in projection (VIP) value. The quality of the OPLS-DA model was assessed using parameters R2Y and Q2 (cum).

Metabolites meeting the following criteria were considered differential: a *P*-value below 0.05, a fold change (FC) above 1.2 or below 0.8, a coefficient of variance below 30% (QC), and a VIP value above 1.0. Online databases such as HMDB, KEGG, and METLIN were consulted to identify potential metabolites, and mass errors were confirmed to be within 5 ppm when compared to MS/MS data. Route enrichment analysis and metabolic pathway analysis were performed using the Metabo Analyst v4.0 online software (http://www.metaboanalyst.ca/).

**Figure 1:**
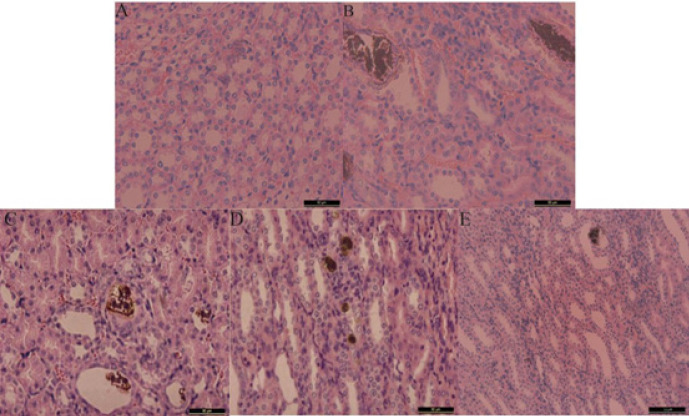
Representative photomicrographs for von Kossa staining of calcium deposition in the cortex and medulla junction of the kidney of rat

**Figure 2:**
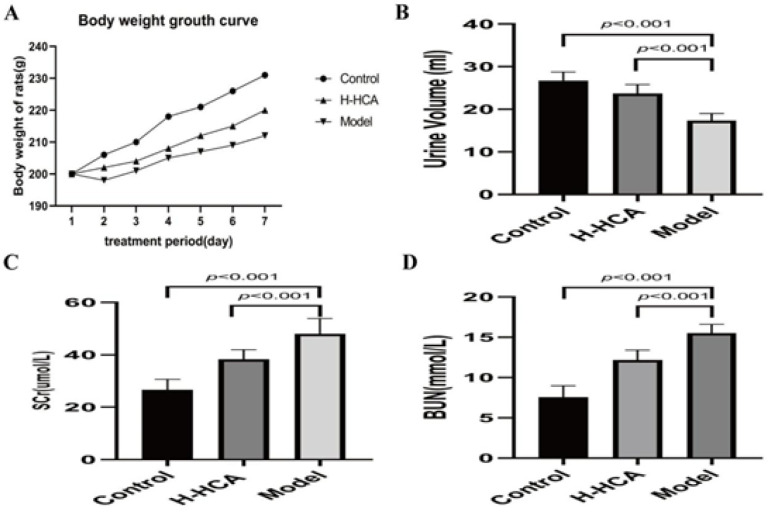
Effects of hydroxycitric acid (HCA) on various physiological and biochemical parameters in rats

(A) Body weight ; (B) Urine volume; (C) Serum creatinine; (D) Blood urea nitrogen

**Figure 3:**
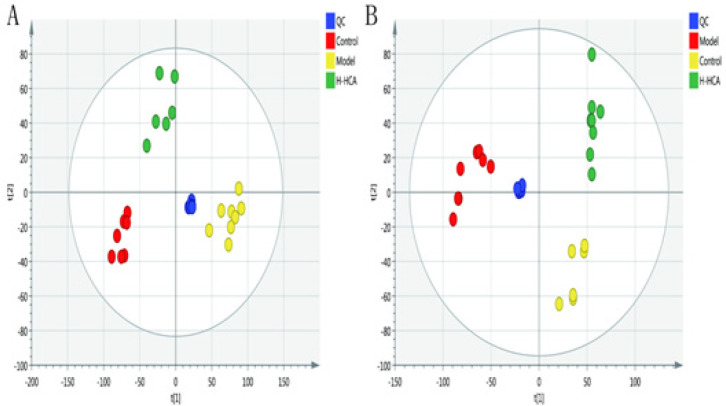
Plots of multivariate statistical analysis based on metabolites and stability obtained from quality control (QC) samples in the positive-ion mode (A) and in the negative-ion mode (B)

**Figure 4:**
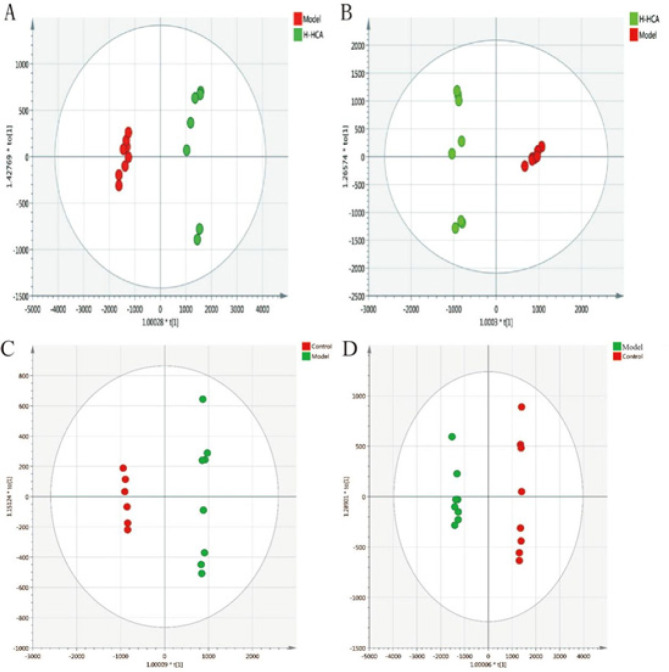
OPLS-A score plots of different groups A and B: OPLS-DA score plots of the model group and the H-HCA-treated group in positive and negative ion mode. C and D: OPLS-DA score plots of the model group and the control group in positive and negative ion mode

**Figure 5:**
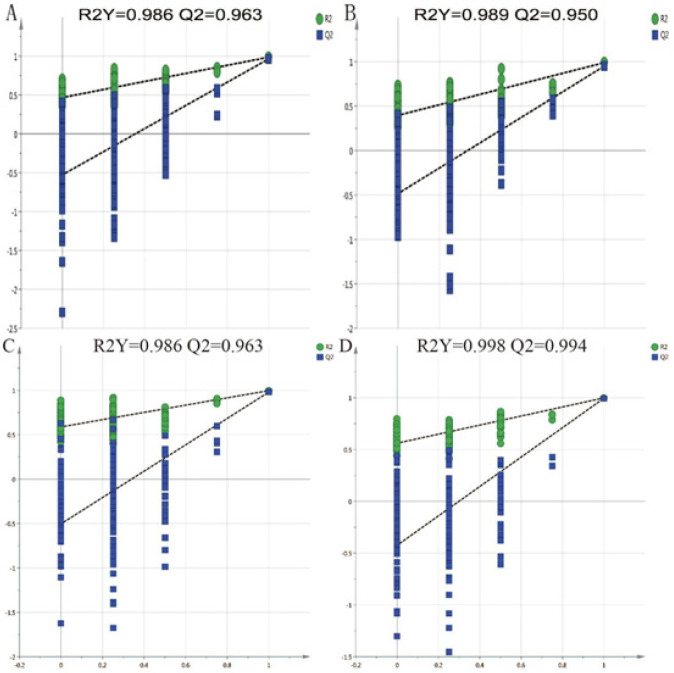
Permutation tests of different groups A and B: Permutation tests of the model group and the H-HCA-treated group in positive and negative ion mode. C and D: Permutation tests of the model group and the control group in positive and negative ion mode

**Figure 6:**
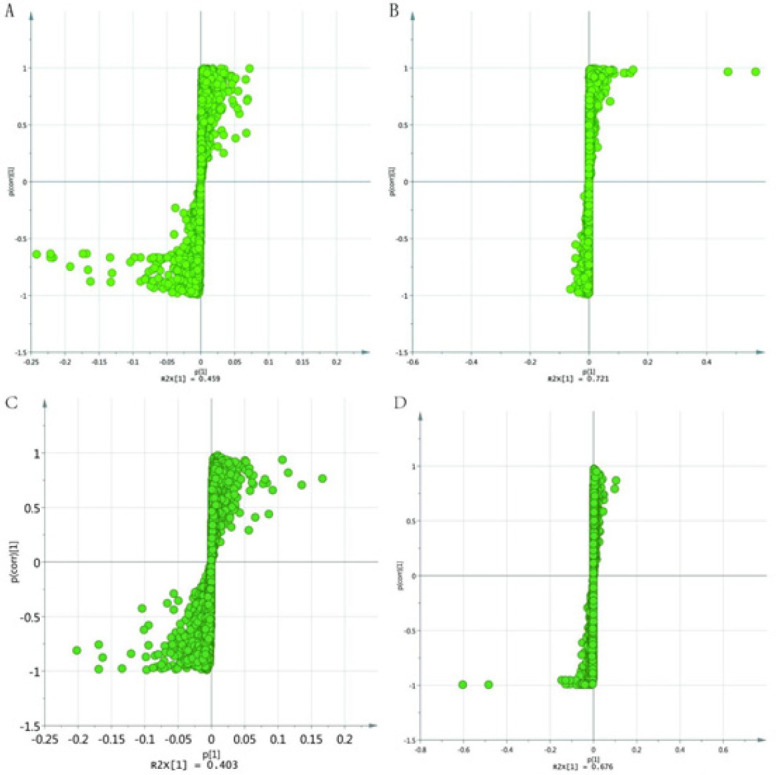
s-plot of different groups

A and B: s-plot of the model group and the H-HCA-treated group in positive and negative ion mode, C and D: s-plot of the model group and the control group in positive and negative ion mode

HCA: hydroxycitric acid, H-HCA: high-dose HCA

**Figure 7:**
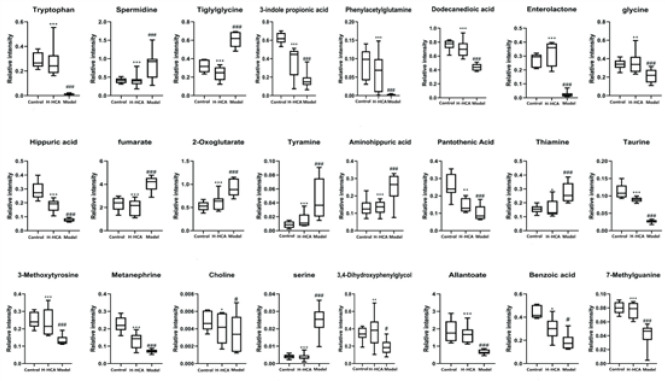
Box plots showing the levels of 24 potential marker metabolites in the control, H-HCA-treated, and model group

*P<0.05, **P<0.01, ***P<0.001 for H-HCA-treated group versus the model group; ^#^*P<*0.05, ^##^*P<*0.01, ^###^*P<*0.001 for the model group versus the control group

HCA: hydroxycitric acid, H-HCA: high-dose HCA

**Figure 8:**
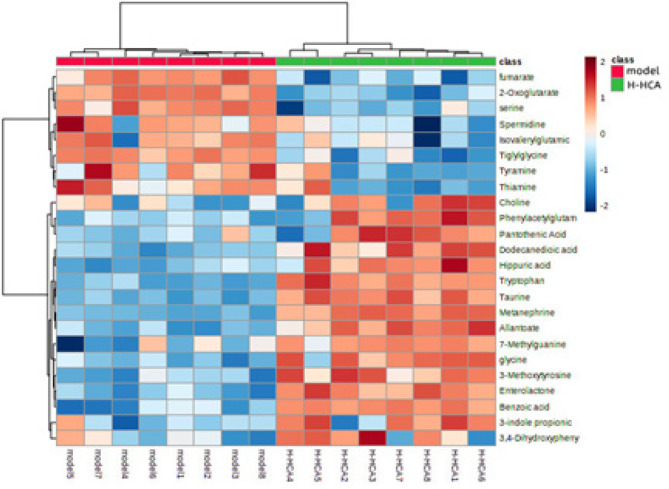
Hierarchical clustering heat-map analysis between the H-HCA-treated group and the model group

HCA: hydroxycitric acid, H-HCA: high-dose HCA

**Figure 9:**
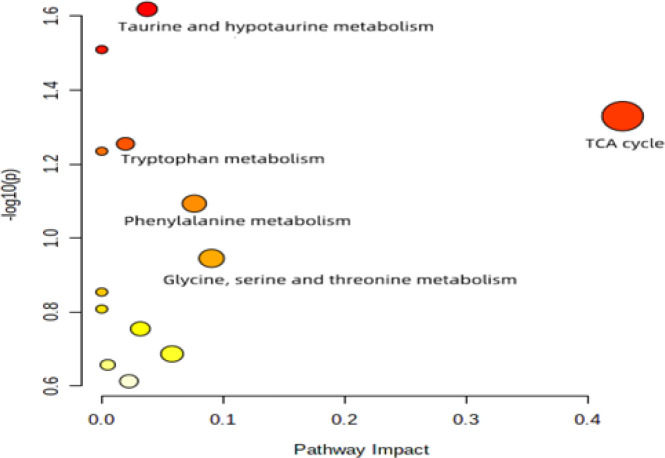
Overview of altered metabolic pathways identified by Metabo Analyst’s Pathway Analysis

**Figure 10:**
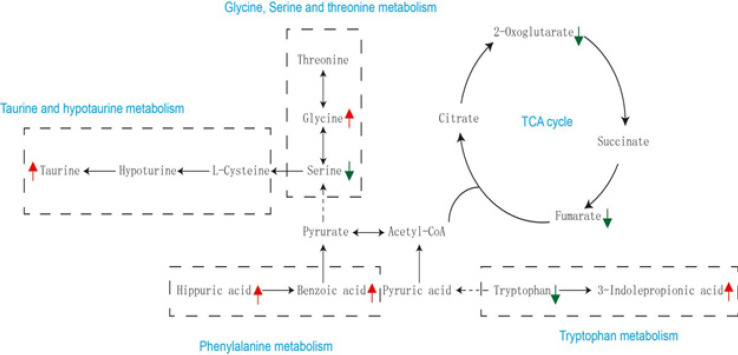
Metabolic pathway networks resulting from hydroxycitric acid (HCA) modulation to the glyoxylate-induced crystal kidney injury

Identified metabolites with a changed concentration in the H-HCA-treated group compared to that in the model group are labeled in red (up-regulated) and green (down-regulated), respectively

**Table 1:** Identification and change trend of biomarkers from urinary metabolic profiling in the control group, high-dose hydroxycitric acid (H-HCA)-treated group, and model group

	No	m/z	Mode	Identification	Formula	Trend (H-HCA treated/Model)	Trend (Control/Model)
ESI+	1	220.3012	[M+H]+	Pantothenic Acid	C9H17NO5	↑	↑
	2	146.0403	[M+H]+	Spermidine	C7H19N3	↓	↓
	3	157.1602	[M+H]+	Tiglylglycine	C7H11NO3	↓	↓
	4	190.0861	[M+H]+	3-indole propionic acid	C11H11NO2	↑	↑
	5	303.0726	[M+H]+	Phenylacetylglutamine	C13H16N2O4	↑	↑
	6	230.3007	[M+H]+	Dodecanedioic acid	C12H22O4	↑	↑
	7	299.1270	[M+H]+	Enterolactone	C18H18O4	↑	↑
	8	76.0214	[M+H]+	glycine	C2H5NO2	↑	↑
	9	180.0578	[M+H]+	Hippuric acid	C9H9NO3	↑	↑
	10	117.0110	[M+H]+	fumarate	C4H4O4	↓	↓
	11	147.0199	[M+H]+	2-Oxoglutarate	C5H6O5	↓	↓
	12	104.0091	[M+H]+	serine	C3H7NO3	↓	↓
	13	195.0765	[M+H]+	Aminohippuric acid	C9H10N2O3	↓	↓
	14	177.0624	[M+H]+	Allantoate	C4H8N4O4	↑	↑
	15	266.1196	[M+H]+	Thiamine	C12H17N4OS	↓	↓
	16	212.0911	[M+Na]+	3-Methoxytyrosine	C10H13NO4	↑	↑
	17	104.1063	[M+Na]+	Choline	C5H14NO	↑	↑
ESI-	18	124.0079	[M-H]-	Taurine	C2H7NO3S	↑	↑
	19	196.0680	[M−H]−	Metanephrine	C10H15NO3	↑	↑
	20	136.0760	[M−H]−	Tyramine	C8H11NO	↓	↓
	21	169.0491	[M−H]−	3,4-Dihydroxyphenylglycol	C8H10O4	↑	↑
	22	203.0821	[M−H]−	Tryptophan	C11H12N2O2	↑	↑
	23	123.0431	[M−H]−	Benzoic acid	C7H6O2	↑	↑
	24	164.0559	[M−H]−	7-Methylguanine	C6H7N5O	↑	↑

**Table 2:** Related pathways of metabolites in the H-HCA-treated group and the model group

Pathway	Total	Hits	p	−log(p)	Impact
Taurine and hypotaurine metabolism	8	1	0.0019	1.6085	0.06196
TCA cycle	20	2	0.0044	1.3783	0.45883
Tryptophan metabolism	41	1	0.0198	1.2181	0.02693
Phenylalanine metabolism	12	1	0.0621	1.0989	0.08632
Glycine, serine and threonine metabolism	34	2	0.1673	0.9522	0.09963

## Results

***Histopathological examination***

The calcium staining images using von Kossa staining are shown in Figure 1. Notably, there were prominent calcium deposits observed in the kidney sections of rats in the model group. Conversely, the renal sections from the HCA-treated group exhibited significantly reduced CaOx deposits compared to the model group, with the most pronounced effect observed at higher HCA doses. Following the results of the dose selection, we determined that the optimal dosage for subsequent metabolomics and biochemistry analyses would be 1 g/kg of HCA treatment.

***Body weight, urine volume, and biochemical changes***

In contrast to the control group, rats in the model group showed significantly lower weight gain, whereas the HCA-treated group tended to recover in body weight. Furthermore, there was a significant increase in urine production among rats treated with HCA. Serum creatinine and blood urea nitrogen levels were significantly higher in the model group compared to the H-HCA-treated group (Figure 2). These findings provide strong evidence supporting the protective effect of HCA against renal stones.

***Metabolic profiling analysis of urine***

The PCA analysis displayed a well-defined clustering pattern among the QC samples, indicating robust stability and reproducibility within our current metabolomics dataset (Figure 3). No outliers were observed in the PCA score plot, and a clear demarcation between the model group and the H-HCA treated group was evident. Subsequently, OPLS-DA was employed to identify potential metabolite biomarkers, demonstrating complete separation between the model group and the H-HCA-treated group, as well as between the control group and the model group (Figure 4). R2Y and Q2 (cum) were used as metrics to evaluate the predictive capability of the model. R2Y and Q2 of the OPLS-DA model as shown in Figure 5, provided further evidence of the model’s strong predictability and validity.

***Identification of potential biomarkers***

The spectral regions that exhibited significant differences between the groups were identified using the S-plot loading plot (Figure 6). Potential biomarkers were considered as candidate metabolites with a VIP value exceeding 1.0. A total of 24 metabolites were identified as potential biomarkers. Among these, 17 metabolites were selected in the positive mode, and 7 metabolites were selected in the negative mode. The up-regulation and down-regulation of these metabolites are presented in Table 1. When using the abundance of these 24 biomarkers as the ordinate and the groups as the abscissa, differences between the groups can be readily discerned (Figure 7).

***Evaluation of the preventive mechanism of HCA***

To visually compare the differential changes in metabolites between the H-HCA-treated group and the model group, a heatmap can be an effective tool. In this study, we utilized the MetaboAnalyst platform to construct a heatmap based on the normalized dataset of the 24 differential metabolites. The heatmap, as shown in Figure 8, clearly illustrates the significant differences in the concentration of these metabolites between the model and the H-HCA-treated group, highlighting distinct expression patterns for the same chemicals.

***Metabolic pathway analysis***

To gain deeper insights into the changes in metabolic pathways resulting from HCA treatment, we conducted a metabolic pathway analysis using the online MetaboAnalyst’s Pathway Analysis tool (Figure 9). The results of the metabolic pathway analysis indicated that HCA treatment primarily impacted the metabolism of glycine, serine, threonine, phenylalanine, the tricarboxylic acid (TCA) cycle, taurine, hypotaurine, and tryptophan. These metabolic pathways, as shown in Table 2, may be key mediators of HCA’s protective effects against CaOx crystal-induced kidney injury.

The primary metabolic pathways associated with the identified biomarkers are presented in Figure 10. Further research into these pathways can provide valuable insights into the therapeutic mechanisms of HCA and how it functions to protect against kidney injury induced by CaOx crystals.

## Discussion

In our study, we induced kidney damage caused by CaOx crystals using glyoxylate as a model. Subsequently, to investigate metabolic pathways responsible for the preventive effects of HCA on kidney damage, we employed UHPLC-Q-TOF/MS-based metabolomics.

Our experimental results confirmed the successful establishment of a CaOx crystal-induced kidney injury model. We have identified 24 metabolites in urine samples that can serve as potential biomarkers for assessing the effectiveness of HCA treatment in the context of CaOx crystal-induced kidney injury. Moreover, by consulting pathway databases, we have constructed a metabolic network that offers valuable insights into the connections between glyoxylate-induced CaOx crystal kidney injury and disruptions in glycine, serine, and threonine metabolism; tryptophan metabolism; TCA cycle; phenylalanine metabolism; as well as taurine and sub-taurine metabolism.

Proteins are essential components of cell membranes, and any disruption in amino acid metabolism can lead to damage to renal tubular epithelial cells (23).In the model group, we observed significant alterations in several amino acids, including tryptophan, glycine, and serine. These findings are consistent with prior research, highlighting the close relationship between renal disorders and changes in the production, breakdown, or excretion of amino acids (24). The pathophysiology of kidney stone formation may indeed be intricately linked to the imbalanced metabolism of amino acids.

In our investigation, we noted that the levels of 3-indole propionic acid (3-IPA) in the H-HCA-treated group were higher than those in the model group. 3-IPA is produced by the gut microbiome through the metabolism of tryptophan and has been shown to offer defense against redox imbalance, inflammation, and cellular lipid damage (25-26). Previous studies have also suggested that 3-IPA may serve as a significant protective factor and a biomarker against the onset of kidney disease (27). 

Glycine has been demonstrated to significantly reduce glycol-induced CaOx crystal deposition in rat kidneys by affecting urinary oxalate and citrate levels (28). It achieves this by decreasing urine oxalate levels through the down-regulation of Slc26a6 expression (29), while simultaneously increasing urine citrate levels by inhibiting Nadc1 expression, thus inhibiting CaOx crystallization (30).

The TCA cycle, a significant aerobic pathway, plays a crucial role in the metabolism of sugars, lipids, and amino acids (31). It is responsible for generating a substantial amount of energy for cellular processes through various oxidative phosphorylation steps. Metabolites like 2-oxoglutarate and fumarate are closely associated with the TCA cycle. After glyoxylate injection, their urinary levels were significantly increased, but following HCA treatment, they were markedly down-regulated. Notably, the reduced urinary fumarate, a key TCA cycle metabolite, may indicate inhibition of the TCA cycle (32). Among hyperoxaluric rats, decreased electron transport chain complex activity and increased oxidative stress were indicative of mitochondrial dysfunction induced by CaOx crystal deposits. In the pathophysiology of kidney stones, mitochondria play a critical role in the TCA cycle, which occurs in the mitochondrial matrix (33).

Taurine, a sulfur-containing amino acid, functions as an endogenous antioxidant and membrane stabilizer in mammalian cells (34). Extensive research has demonstrated its protective effects against various forms of kidney injury, including ischemia/reperfusion injury, hyperglycemia, oxidative stress, and xenobiotics (35, 36). It has been demonstrated that taurine therapy in rat models of nephrolithiasis reduces tubular oxidative damage through a mitochondrial-linked pathway (37, 38). The observed increase in urine taurine levels in rats treated with HCA suggests that the induction of taurine may contribute to the preventive effect of HCA against CaOx crystal-induced renal damage. Given the wealth of studies highlighting its beneficial effects on kidney function, taurine holds promise as a potential treatment for kidney diseases.

Compared to the H-HCA-treated group, the concentrations of hippuric and benzoic acid were significantly lower in the model group. Furthermore, in rats induced to develop kidney stones experimentally, a decrease in the urinary levels of benzoic acid was also observed (39). These compounds are associated with the metabolism of phenylalanine. Previous studies have suggested that hippuric acid can act as an inhibitor of CaOx urolithiasis by forming a complex with calcium ions, known as hippurate. This complex formation reduces urinary calcium levels and inhibits the formation of CaOx (40). Additionally, a previous study has recommended monitoring the urinary excretion of hippuric acid as a biomarker for assessing dietary fruit and vegetable intake (41,42). Furthermore, it has been proposed that the urinary hippuric acid level should exceed 300 mg per 24 hours (43).

## Conclusion

In summary, the present study has substantiated HCA’s preventive properties against CaOx crystal-induced renal damage in rats. The administration of HCA led to a reduction in CaOx crystal deposition and an improvement in renal function. Employing a metabolomics-based approach to investigate urine metabolic profiles, we discovered that HCA administration reversed the metabolic abnormalities induced by CaOx crystal exposure. The identification of 24 altered biomarkers suggests that HCA’s effects on CaOx crystal nephropathy may be primarily attributed to the restoration of disturbances in glycine, serine, and threonine metabolism; phenylalanine metabolism; TCA cycle; taurine and hypotaurine metabolism; and tryptophan metabolism. Urine metabolomics serves as a powerful tool for delving into the mechanisms underlying the therapeutic efficacy of HCA.
